# Supplementary figures and images for: Compilation of reported protein changes in the brain in Alzheimer’s disease
Source: Nat Commun. 2023 Jul 25;14:4466. doi: 10.1038/s41467-023-40208-x (PMC10368642; doi:10.1038/s41467-023-40208-x)

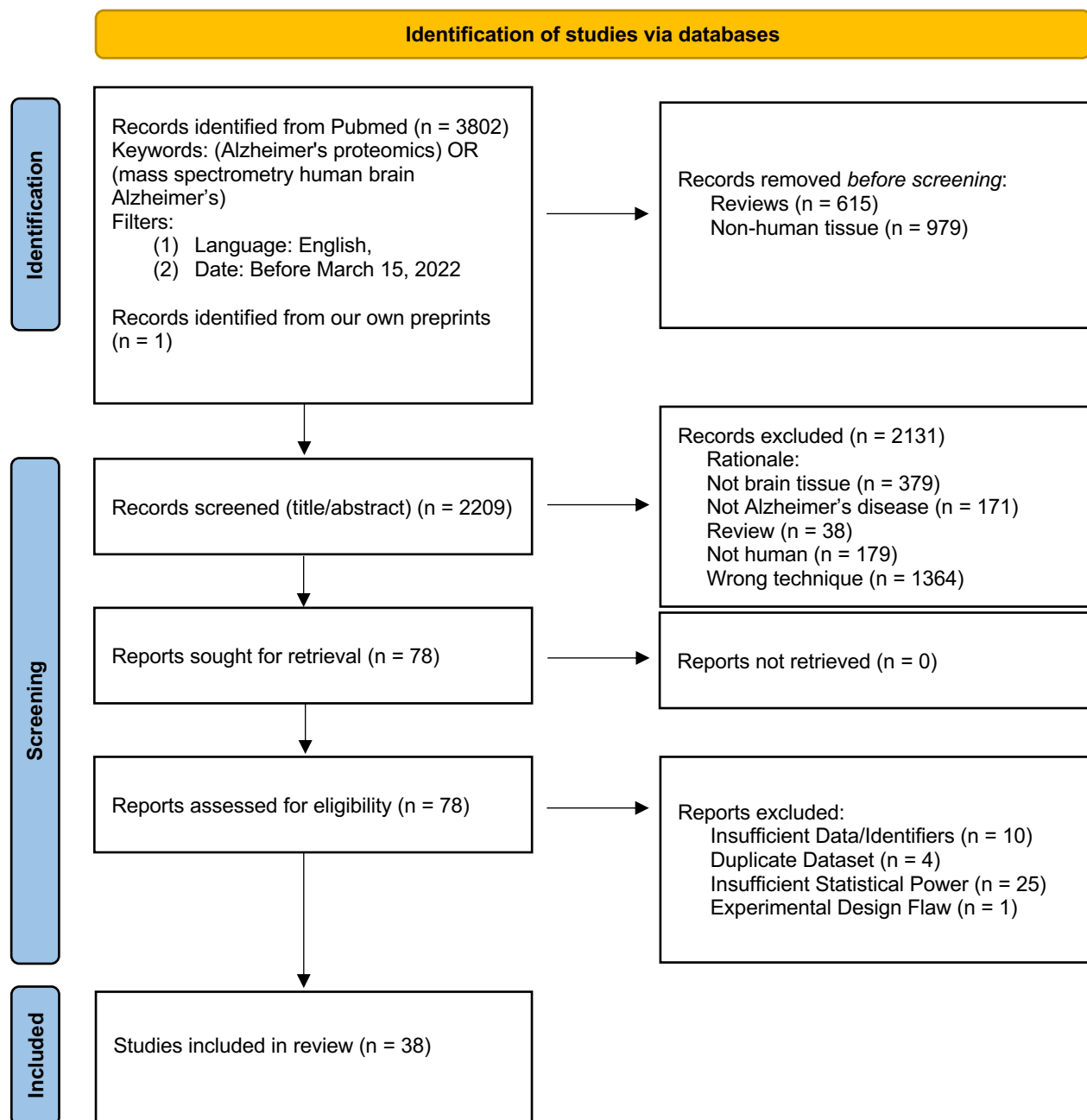

**Supplementary Figure 1: PRISMA flow diagram describing systematic review process.**

Supplement: Supplementary file 1 — Supplementary Information [file 41467_2023_40208_MOESM1_ESM.pdf]
