# Supplementary material for: Compilation of reported protein changes in the brain in Alzheimer’s disease
Source: Nat Commun. 2023 Jul 25;14:4466. doi: 10.1038/s41467-023-40208-x (PMC10368642; doi:10.1038/s41467-023-40208-x)
Supplement: Supplementary file 3 — Description of Additional Supplementary Files [file 41467_2023_40208_MOESM3_ESM.pdf]

Description of Additional Supplementary Files

File Name: Supplementary Data 1

Description: Complete NeuroPro dataset

File Name: Supplementary Data 2

Description: 848 proteins significantly altered in ≥5 bulk tissue studies

File Name: Supplementary Data 3

Description: Gene ontology enrichments for Cellular Compartment (CC) and Biological Process (BP) for the 848 proteins significantly altered in ≥5 bulk tissue studies

File Name: Supplementary Data 4

Description: Gene Ontology enrichments for Cellular Compartment (CC) of proteins enriched in NFTs or Plaques

File Name: Supplementary Data 5

Description: Proteins altered at different clinical stages of AD

File Name: Supplementary Data 6

Description: Gene ontology enrichments for Cellular Compartment (CC) used to generate the enrichment plot in Figure 5B

File Name: Supplementary Data 7

Description: Brain region specific protein changes in advanced AD bulk tissue studies

File Name: Supplementary Data 8

Description: Protein changes present in both early clinical stages of AD (preclinical AD/MCI) and in resistant brain regions

File Name: Supplementary Data 9

Description: Proposed staging of Alzheimer's disease protein changes in human brain tissue

File Name: Supplementary Data 10

Description: Proteins with inconsistent directional differences between proposed AD phases
