# Supplementary material for: Compilation of reported protein changes in the brain in Alzheimer’s disease
Source: Nat Commun. 2023 Jul 25;14:4466. doi: 10.1038/s41467-023-40208-x (PMC10368642; doi:10.1038/s41467-023-40208-x)
Supplement: Supplementary file 5 — Reporting Summary [file 41467_2023_40208_MOESM5_ESM.pdf]

nature portfolio

Corresponding author(s): Eleanor Drummond

Last updated by author(s): Jul 5, 2023

Reporting Summary

Nature Portfolio wishes to improve the reproducibility of the work that we publish. This form provides structure for consistency and transparency in reporting. For further information on Nature Portfolio policies, see our Editorial Policies and the Editorial Policy Checklist.

Statistics

For all statistical analyses, confirm that the following items are present in the figure legend, table legend, main text, or Methods section.

n/a	Confirmed	
☐	☒	The exact sample size (*n*) for each experimental group/condition, given as a discrete number and unit of measurement
☐	☒	A statement on whether measurements were taken from distinct samples or whether the same sample was measured repeatedly
☐	☒	The statistical test(s) used AND whether they are one- or two-sided<br>*Only common tests should be described solely by name; describe more complex techniques in the Methods section.*
☐	☒	A description of all covariates tested
☐	☒	A description of any assumptions or corrections, such as tests of normality and adjustment for multiple comparisons
☐	☒	A full description of the statistical parameters including central tendency (e.g. means) or other basic estimates (e.g. regression coefficient) AND variation (e.g. standard deviation) or associated estimates of uncertainty (e.g. confidence intervals)
☐	☒	For null hypothesis testing, the test statistic (e.g. *F*, *t*, *r*) with confidence intervals, effect sizes, degrees of freedom and *P* value noted<br>*Give P values as exact values whenever suitable.*
☐	☒	For Bayesian analysis, information on the choice of priors and Markov chain Monte Carlo settings
☐	☒	For hierarchical and complex designs, identification of the appropriate level for tests and full reporting of outcomes
☐	☒	Estimates of effect sizes (e.g. Cohen's *d*, Pearson's *r*), indicating how they were calculated

Our web collection on statistics for biologists contains articles on many of the points above.

Software and code

Policy information about availability of computer code

Data collection	Studies were identified using PubMed. If required, UniProt accession IDs were generated from alternative published accession IDs using UniProt (Release 2022_1).
Data analysis	R v4.0.2 or higher (https://www.r-project.org/);<br>RStudio 2022.07.2+576.pro12 or higher (https://www.rstudio.com/products/rstudio/download/);<br>STRING v11.5 (https://string-db.org/);<br>UniProt release 2022_01 or later (https://www.uniprot.org/);<br>MitoCoP: Morgenstern, M. et al (2021) Cell Metab 33(12):2464-2483. DOI: 10.1016/j.cmet.2021.11.001<br>SynGo release "20210225" (https://www.syngoportal.org/);<br>Cytoscape v3.9.1 or higher (https://cytoscape.org/);<br>Adobe Illustrator v27.1.1 or higher (https://www.adobe.com/products/illustrator/);<br>BioRender (https://www.biorender.com/);

For manuscripts utilizing custom algorithms or software that are central to the research but not yet described in published literature, software must be made available to editors and reviewers. We strongly encourage code deposition in a community repository (e.g. GitHub). See the Nature Portfolio guidelines for submitting code & software for further information.

Data

Policy information about availability of data

All manuscripts must include a data availability statement. This statement should provide the following information, where applicable:

- Accession codes, unique identifiers, or web links for publicly available datasets
- A description of any restrictions on data availability
- For clinical datasets or third party data, please ensure that the statement adheres to our policy

All data used in this study was obtained from prior publications that were obtained from Pubmed (listed in detail with accompanying citations in Table 1). All data used in this study is available on the open-access NeuroPro website: https://neuropro.biomedical.hosting. All data used in sub-analyses are included in full in the supplementary tables of this manuscript.

Research involving human participants, their data, or biological material

Policy information about studies with human participants or human data. See also policy information about sex, gender (identity/presentation), and sexual orientation and race, ethnicity and racism.

Reporting on sex and gender	This was not applicable to the current study as no new data was generated. Our analysis relied solely on previously published data.
Reporting on race, ethnicity, or other socially relevant groupings	This was not applicable to the current study as no new data was generated. Our analysis relied solely on previously published data.
Population characteristics	This was not applicable to the current study as no new data was generated. Our analysis relied solely on previously published data.
Recruitment	This was not applicable to the current study as no new data was generated. Our analysis relied solely on previously published data.
Ethics oversight	This was not applicable to the current study as no new data was generated. Our analysis relied solely on previously published data.

Note that full information on the approval of the study protocol must also be provided in the manuscript.

Field-specific reporting

Please select the one below that is the best fit for your research. If you are not sure, read the appropriate sections before making your selection.

☒ Life sciences ☐ Behavioural & social sciences ☐ Ecological, evolutionary & environmental sciences

For a reference copy of the document with all sections, see nature.com/documents/nr-reporting-summary-flat.pdf

Life sciences study design

All studies must disclose on these points even when the disclosure is negative.

Sample size	No sample size calculation was performed. Sample size was chosen based on available data.
Data exclusions	No data was excluded in the compilation of previously published studies.<br>One directional outlier was excluded when defining a protein as consistently increased or decreased in advanced Alzheimer's disease only in the specific instance where the protein was consistently altered in the same direction in at least 4 other studies and only one directional outlier was present. This was done to accommodate expected variation due to a range of brain regions, fractions, and disease stages being included in this analysis.
Replication	This study combines data from 38 previously published studies and specifically reports on the reproducibility of their individual findings.
Randomization	No sample allocation into experimental groups was performed in this study as it was a compilation of previously published studies; all sample allocation into experimental groups was performed in these prior datasets, which we relied on for our group definition in the current study.
Blinding	Blinding was not relevant to this study as there was no intervention being examined.

Reporting for specific materials, systems and methods

We require information from authors about some types of materials, experimental systems and methods used in many studies. Here, indicate whether each material, system or method listed is relevant to your study. If you are not sure if a list item applies to your research, read the appropriate section before selecting a response.

Materials & experimental systems

n/a	Involved in the study
☒	☐ Antibodies
☒	☐ Eukaryotic cell lines
☒	☐ Palaeontology and archaeology
☒	☐ Animals and other organisms
☒	☐ Clinical data
☒	☐ Dual use research of concern
☒	☐ Plants

Methods

n/a	Involved in the study
☒	☐ ChIP-seq
☒	☐ Flow cytometry
☒	☐ MRI-based neuroimaging
